# Bilateral anterior capsulotomy enhances medication compliance in patients with epilepsy and psychiatric comorbidities

**DOI:** 10.1111/cns.13118

**Published:** 2019-03-13

**Authors:** Peng Huang, Deng Zheng‐Dao, Bo‐Min Sun, Yi‐Xin Pan, Jing Zhang, Tao Wang, Wei Liu, Hai‐Yan Jin, Shi‐Kun Zhan

**Affiliations:** ^1^ Department of Functional Neurosurgery, Ruijin Hospital Shanghai Jiao Tong University School of Medicine Shanghai China; ^2^ Department of Psychiatry, Ruijin Hospital Shanghai Jiao Tong University School of Medicine Shanghai China

**Keywords:** epilepsy, epilepsy psychiatric comorbidity, psychosurgery, stereotactic bilateral anterior capsulotomy

## Abstract

**Objectives:**

Patients with epilepsy and refractory comorbid psychiatric disorders often experience functional impairments and a lower quality of life as well as showing a lack of compliance with anti‐epileptic medication regimens. We reasoned that widespread clinical benefits could be gained if the psychiatric comorbidities among these patients were reduced. In this study, we assessed the utility of anterior capsulotomy in managing medication‐refractory comorbid psychotic symptoms and aggression in patients with epilepsy.

**Methods:**

In this retrospective case series, we evaluated the clinical outcomes of 13 epilepsy patients with severe psychiatric comorbidities who had received bilateral anterior capsulotomy. Clinical outcome assessments were performed at 1 week, 6 months, 1 year, and several years after surgery focusing on: (a) severity of psychotic symptoms, as assessed by the 18‐item Brief Psychiatric Rating Scale and the Positive and Negative Syndrome Scale; (b) severity of impulsivity and aggression, measured by the Barratt Impulsiveness Scale‐11 and the Buss‐Perry Aggression Scale; and (c) social function and quality of life, assessed by the Social Disability Screening Scale and the Quality of Life in Epilepsy.

**Results:**

After anterior capsulotomy, patients displayed significant improvements of psychotic symptoms, as well as of impulsivity and aggression, along with improvements of social function and quality of life. The clinical benefits to patients were evident within 6 months after surgery and remained stable or continued to improve at a much slower rate thereafter. Furthermore, after anterior capsulotomy all patients complied with epilepsy interventions that they did not comply with prior to surgery. No significant side effects or complications occurred during the study.

**Conclusion:**

Anterior capsulotomy seems to be a safe and effective treatment for epilepsy patients with otherwise intractable comorbid psychotic symptoms and aggression. Moreover, this neurosurgical treatment may improve the patients' social function, quality of life, and compliance with anti‐epilepsy medication regimens.

## 1 INTRODUCTION

Epilepsy encompasses a group of different syndromes characterized by recurrent unprovoked seizures. This neurological disease is relatively common, afflicting at least 70 million people in the world.^1^, ^2^ Epilepsy is complex in its etiology and clinical manifestations.^3^ Compared to the general population, patients with epilepsy have a higher risk for suicidal ideation and psychiatric disorders.^4^, ^5^ In particular, it is estimated that about 46% of patients with epilepsy has a comorbid anxiety disorder. Moreover, about 25% of epilepsy patients suffers from depression,^6^, ^7^ and 5%‐14% attempts or commits suicide.^8^ Additionally, 4%‐38% patients with epilepsy displays psychotic symptoms either with or without aggression.^9^ The presence of psychiatric comorbidities among patients with epilepsy poses a major clinical challenge because it adversely affects the patients' general functioning and quality of life, as well as their compliance with anti‐epileptic medication regimens and other therapeutic interventions.

Patients with epilepsy who also present with psychotic symptoms and aggression represent a particularly challenging patient group to treat and care for. First, these patients typically receive antipsychotic medications to alleviate the comorbid “positive” (eg, delusions, hallucinations) and “negative” (eg, apathy, blunted affect) symptoms of schizophrenia. Antipsychotic medications, however, may not always be effective and may even exacerbate seizure frequency and severity.^10^ Second, due to the nature of their psychiatric comorbidities, these patients often fail to comply with prescribed anti‐epileptic medication regimens. This lack of compliance also applies to other therapeutic interventions offered, including neurophysiologic (eg, EEG, MEG, MRI) assessments required for seizure monitoring or surgery. Thus, the presence of severe medication‐refractory psychiatric comorbidities can greatly disrupt epilepsy treatment for this group of patients. In the present study, we assessed the utility of anterior capsulotomy in improving their mental health status and compliance with anti‐epileptic medication regimens.

Anterior capsulotomy was first introduced by Talairach, and refined by Leksell for treating various psychiatric diseases in the early 1950s.^11^, ^12^ Since then, anterior capsulotomy has been mainly applied to the treatment of severe cases of refractory obsessive‐compulsive disorder.^13^ In recent years, however, anterior capsulotomy has also been applied successfully to the treatment of other psychiatric disorders, including depression^14^ and schizophrenia.^15^ Accordingly, in this retrospective case series we assessed the utility of anterior capsulotomy in managing refractory comorbid psychotic symptoms and aggression in several patients with epilepsy. Additionally, we examined the effects of anterior capsulotomy on patients' psychosocial function and quality of life. We hypothesized that anterior capsulotomy could be effective in improving both the patients' mental health status and their compliance with anti‐epileptic medication regimens.

## 2 MATERIALS AND METHODS

### 2.1 Participants

Study participants consisted of 13 patients (eight male and five female, age at surgery: mean = 30, SD = 12 years) with epilepsy and severe psychiatric comorbidities. The patients had received magnetic resonance imaging (MRI)‐guided stereotactic bilateral anterior capsulotomy at Ruijin Hospital between January 2009 and December 2015. All patients had been diagnosed with epilepsy and treated with regular anti‐epileptic drugs (AEDs). Psychiatric comorbidity consisted mainly of positive psychotic symptoms, as well as of aggression and excessive impulsivity, followed by anxiety, depression, and intellectual disability (Table 1). The study was approved by the Ethics Committee of Ruijin Hospital. Written informed consent was obtained from each patient. All patients fulfilled the surgical inclusion criteria outlined below.

**Table 1:** Patients demographics (N = 13)

	Gender	Psychiatric manifestations	Neuroleptic medications （pre/postoperation)	Age at surgery	Disease course for epileptic psychiatric comorbidities (Y)	Follow‐up (Mo)
Patient 1	Female	Aggressive behaviors, impulsive behaviors, intellectual disability, emotional depression	None/none	42	3	21
Patient 2	Female	Aggressive behaviors, impulsive behaviors	Olanzapine^1^, Aripiprazole^2^, Sertraline^3^/Olanzapine^1^, Aripiprazole^2^, Sertraline^3^	42	4	24
Patient 3	Male	Aggressive behaviors, impulsive behaviors, hallucinations	Aripiprazole^2^/Aripiprazole^2^	48	6	51
Patient 4	Female	Aggressive behaviors, impulsive behaviors	Clozapine^4^/Clozapine^4^	21	11	54
Patient 5	Male	Aggressive behaviors	Risperidone^5^, Citalopram/Risperidone^5^, Citalopram^6^	25	2	57
Patient 6	Female	Persecutory delusion	Clozapine. Risperidone^5^/Clozapine^4^. Risperidone^5^	49	5	38
Patient 7	Male	Aggressive behaviors, social anxiety, intellectual disability	none/Olanzapine^1^	19	2	42
Patient 8	Male	Aggressive behaviors	none/Aripiprazole	13	2	45
Patient 9	Male	Aggressive behaviors, impulsive behaviors	Clozapine^4^/none	16	10	35
Patient 10	Male	Social anxiety, emotional depression	Aripiprazole^2^, Clozapine^4^/none	27	10	25
Patient 11	Female	Hallucinations, persecutory delusion	Risperidone^5^, Aripiprazole^2^/Risperidone^5^	25	18	64
Patient 12	Male	Aggressive behaviors, emotional depression, social anxiety, hallucinations,	Olanzapine^1^/Olanzapine^1^	34	6	69
Patient 13	Male	Aggressive behaviors, impulsive behaviors	Risperidone^5^, Olanzapine^1^/Risperidone^5^	31	2	70
Average ± SD	/	/	/	30 ± 12	6 ± 5	46 ± 17

### 2.2 Criteria for surgery

Patients who underwent the operation all satisfied the following criteria: (A) presence of severe psychiatric comorbidity, as indexed by a total score of 35 or more on the 18‐item Brief Psychiatric Rating Scale (BPRS, item score 1‐7); (B) presence of severe impulsivity and aggressiveness, as assessed by a psychiatrist; (C) history of no clinical response to at least two types of antipsychotic medications; (D) history of poor compliance with epilepsy therapy and clinical assessments (eg, EEG, MEG, MRI, PET); (E) involvement of family members who were significantly affected by the patient's psychiatric comorbidity; (F) involvement of a family member who, in addition to the patient self, provided written informed consent.

### 2.3 Clinical outcome assessment

Each patient was clinically evaluated by a neurologist and a psychiatrist at the time of surgery and at 1‐week, 6‐month, 1‐year, and long‐term (mean = 46 months, SD = 17) follow‐up. One patient was lost to 1‐year follow‐up. Patients were evaluated using well‐validated clinical rating scales focusing on: (a) severity of positive and negative psychotic symptoms, as measured by the 18‐item Brief Psychiatric Rating Scale and Positive and Negative Syndrome Scale (PANSS); (b) aggressiveness and impulsivity, as measured by the Barratt Impulsiveness Scale‐11 (BIS‐11; three subscales: no‐planning, cognition, and motor) and Buss‐Perry Aggression Scale; (c) social impairment, assessed by the Social Disability Screening Scale (SDSS); and (4) quality of life, as assessed by Quality of Life in Epilepsy (QOLIE‐31), at 1‐year follow‐up.

### 2.4 Surgical procedure

**Figure 1:**
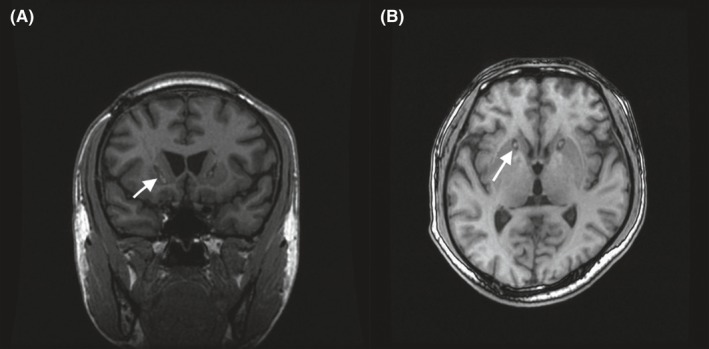
6‐A: T1‐weighted coronal sequence. 6‐B: T1‐weighted axial sequence. Postoperative magnetic resonance images showing the lesions located in the anterior capsule. A, T1‐weighted coronal sequence, and (B) T1‐weighted axial sequence showing the lesions (white arrows)

The capsulotomy procedure used in this study followed the protocol described by Loughman et al^16^ Specifically, a Leksell stereotactic frame (Elekta Inc, Stockholm, Sweden) was mounted on the patient's head under local anesthesia or mild sedation. After placement of the frame, the patient underwent a preoperative MRI scan at 1.5 Tesla (GE Healthcare, Madison, WI, USA). Visualizing the internal capsule on stereotactic MRIs enabled the identification of the target. The target was 15‐17 mm anterior to the anterior commissure (AC), 15‐17 mm lateral to the midline, and 2‐4 mm under the AC‐posterior commissure (PC) line. After calculating the stereotactic target and designing the trajectory, bilateral burr holes were made anterior to the coronal suture based on the measured entrance trajectory. The procedure was performed under general anesthesia after dural opening and cauterization of the pia‐arachnoid. A 2‐mm (diameter) and 3‐mm uninsulated tip radiofrequency electrode (Radionics, Burlington, MA, USA) was used for impedance measurement, followed by a stimulation test and lesion. The radiofrequency lesions were made at 80°C for 60 seconds. The first lesion was located 3‐4 mm below the AC‐PC line, and the total lesioned length was 14 mm bilaterally. A representative MRI performed after this procedure is shown in Figure 1.

### 2.5 Drug therapy

All patients were treated with conventional AEDs. All patients except one also took antipsychotic medications (Table 1). Dose and type of AEDs were not adjusted after surgery. The dose of postoperative psychiatric drugs was tapered gradually according to patient remission status; the psychiatric status of some patients improved to such an extent that they no longer needed medication.

### 2.6 Statistical analysis

Firstly, we used Kolmogorov‐Smirnov tests for normality. These tests were not significant (*P* > 0.05), indicating that the outcome variables did not deviate significantly from normality and that the relationships between the variables were homoscedastic. Subsequently, we performed several one‐way repeated‐measures multivariate analyses of variance, including time as a within‐subjects factor (five levels: baseline, 1‐week, 6‐month, 12‐month, last follow‐up), to assess whether significant differences in the outcome measures existed between the five periods. Follow‐up pairwise comparisons, using post hoc Bonferroni‐corrected paired‐samples t‐tests, were employed to determine whether the outcome measures at baseline were significantly different from those at the three follow‐up periods. For the QOLIE‐31 data, we used Wilcoxon signed‐ranks tests to compare the patients' quality of life data before and after surgery. All statistical analyses were performed using IBM SPSS (version 23).

## 3 RESULTS

### 3.1 Psychotic symptoms

**Figure 2:**
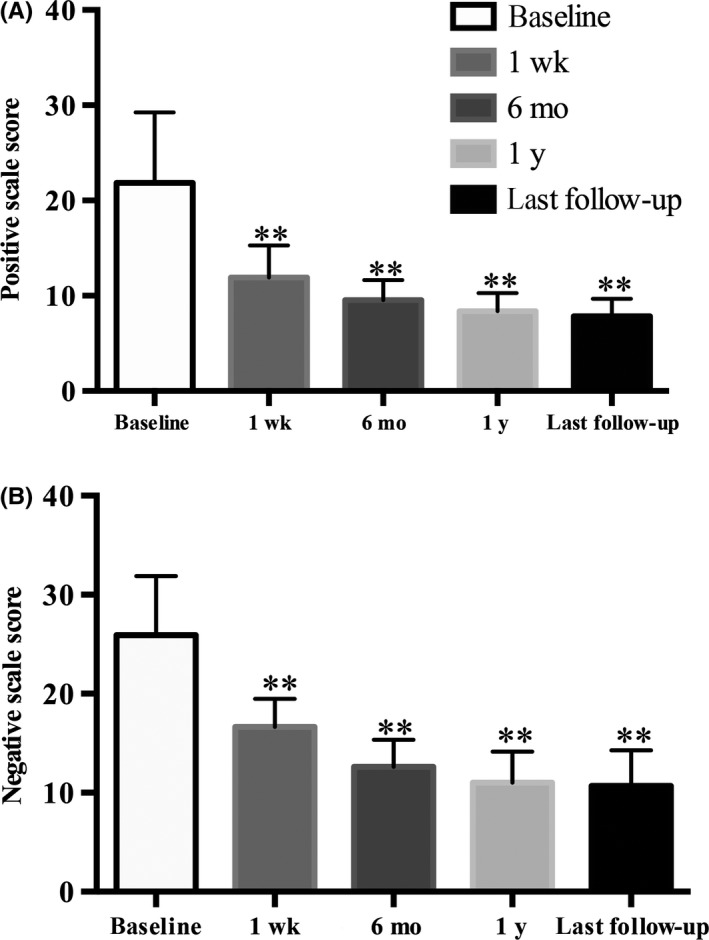
This column bar graphs show the positive and negative scores at the postoperative four follow‐ups. **P ≤ 0.001 vs baseline; (A) Positive scale scoring, (B) Negative scale scoring

After surgery, the severity of both positive symptoms and negative symptoms, as indexed by the PANSS scale scores, was significantly reduced among the patients with epilepsy (Table 2, Figure 2). For example, the positive scale score was improved by 56% and the negative scale score improved by 51% within 6 months after surgery. After 6 months, both types of psychotic symptoms continued to improve, though at a much slower rate, across the follow‐up assessments. At the final follow‐up, positive and negative symptoms were improved by, respectively, 64% and 59% relative to baseline.

**Table 2:** Clinical assessments of patients (N = 13) before and after surgery

	Baseline	1 wk (improvement%)	6 mo (improvement%)	12 mo (improvement%)	Last follow‐up(improvement%)	*P*‐value			
						Baseline vs 1 wk	Baseline vs 6 mo	Baseline vs 12 mo	Baseline vs last follow‐up
Positive scale Scoring	21.9 ± 7.40	11.9 ± 3.35	9.54 ± 2.11	8.38 ± 1.89	7.83 ± 1.85	＜0.001	＜0.001	＜0.001	＜0.001
Negative scale Scoring	25.9 ± 5.95	16.6 ± 2.84	12.6 ± 2.72	11.00 ± 3.16	10.7 ± 3.60	＜0.001	＜0.001	＜0.001	＜0.001
Cognitive Impulsiveness Scoring	32.3 ± 7.25	11.5 ± 3.89	8.08 ± 3.56	7.12 ± 4.32	7.08 ± 4.24	＜0.001	＜0.001	＜0.001	＜0.001
No‐Planning Impulsiveness Scoring	29.2 ± 6.41	11.0 ± 4.63	9.42 ± 3.56	8.85 ± 3.48	8.54 ± 3.61	＜0.001	＜0.001	＜0.001	＜0.001
Motor Impulsiveness Scoring	50.9 ± 9.66	27.3 ± 4.14	5.58 ± 4.69	5.00 ± 4.89	5.42 ± 4.87	＜0.001	＜0.001	＜0.001	＜0.001
Social Disability Screening Schedule	12.6 ± 3.75	7.38 ± 2.63	5.54 ± 2.85	5.53 ± 3.43	5.0 ± 3.54	＜0.001	0.003	0.005	0.004
Buss‐perry Scale	77.7 ± 8.47	60.9 ± 6.09	51.1 ± 9.00	48.8 ± 10.79	48.2 ± 12.3	＜0.001	＜0.001	＜0.001	＜0.001

### 3.2 Impulsivity and aggression

**Figure 3:**
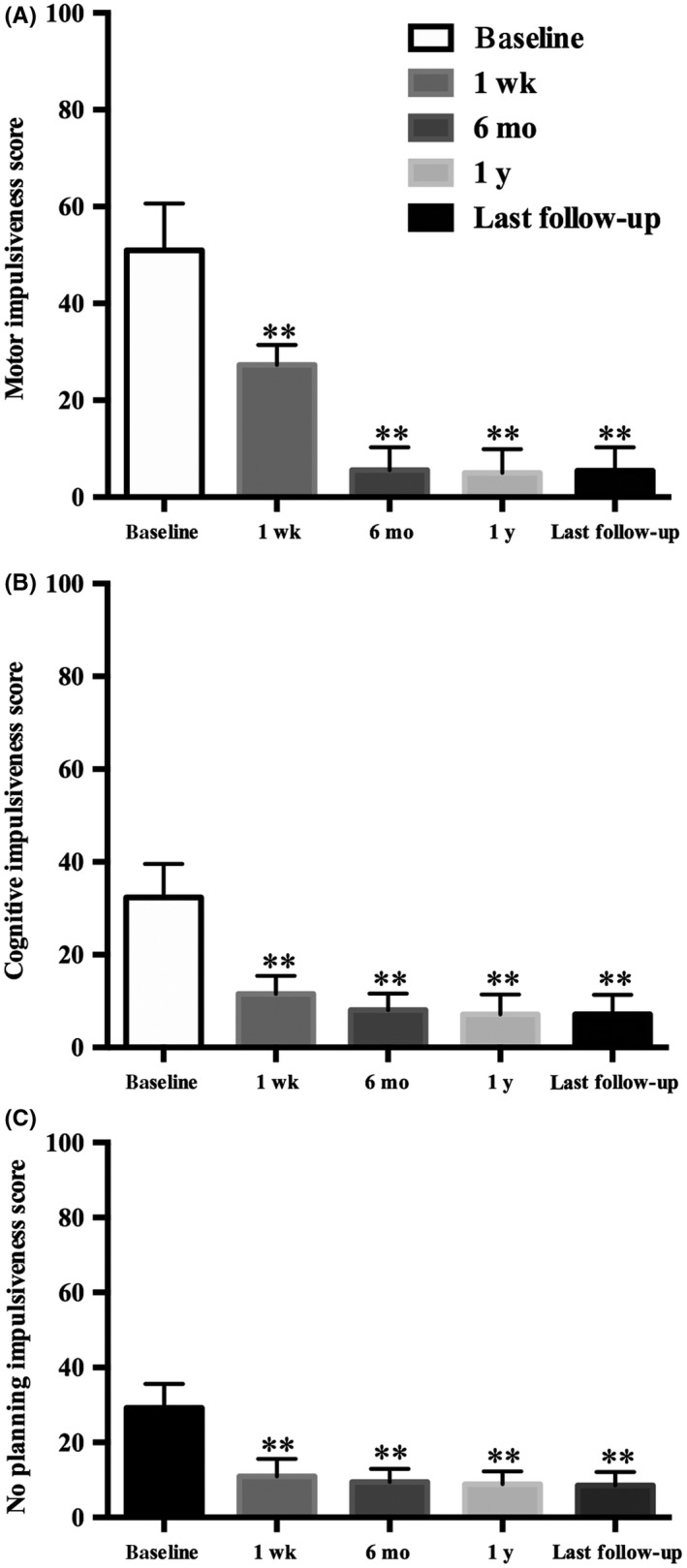
This column bar graphs show the Barratt Impulsiveness Scale‐11 (BIS‐11 [no‐planning, cognition, and motor subscales]) at the postoperative four follow‐ups. **P ≤ 0.01 vs baseline; (A) Motor Impulsiveness scoring, (B) Cognitive impulsiveness scoring, (C) No‐planning impulsiveness scoring

Similarly, patients exhibited a significant reduction in their level of impulsivity, as measured by the BIS‐11, across the follow‐up periods (Table 2, Figure 3). As indexed by the BIS‐11 subscale scores, all three aspects of impulsivity were substantially improved following surgery. Although not formally tested, the motor aspect exhibited a larger improvement than the other aspects of impulsivity. For example, within 6 months after surgery, the patients' levels of motor impulsiveness, cognitive impulsiveness, and no‐planning impulsiveness were reduced by 89%, 75%, and 68%, respectively. After 6 months, the levels of impulsivity remained relatively stable across the follow‐up periods.

**Figure 4:**
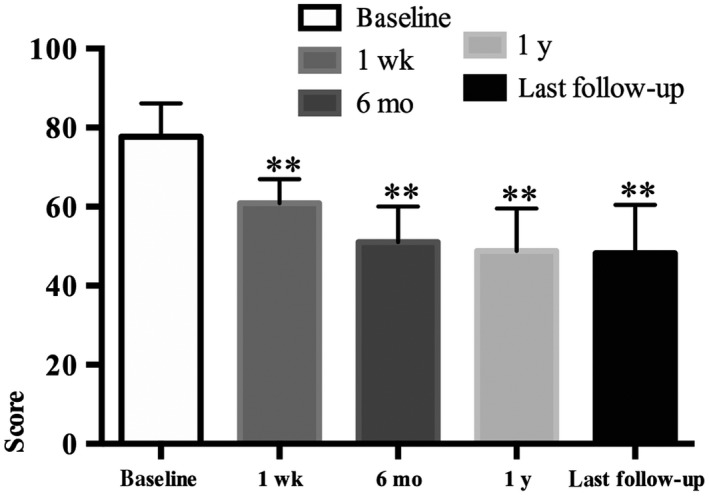
This column bar graph shows the Buss‐Perry Scale at the postoperative four follow‐ups. **P ≤ 0.001 vs baseline

The patients' level of aggressiveness, assessed by the Buss‐Perry Scale, was also significantly reduced after surgery (Table 2, Figure 4). However, the reduction of aggression was smaller than the reduction of impulsivity following surgery. For example, at 6‐month follow‐up, the patients' level of aggression was reduced by 34% relative to baseline level. After 6 months, aggression levels did not further change over the follow‐up periods.

### 3.3 Social function and quality of life

**Figure 5:**
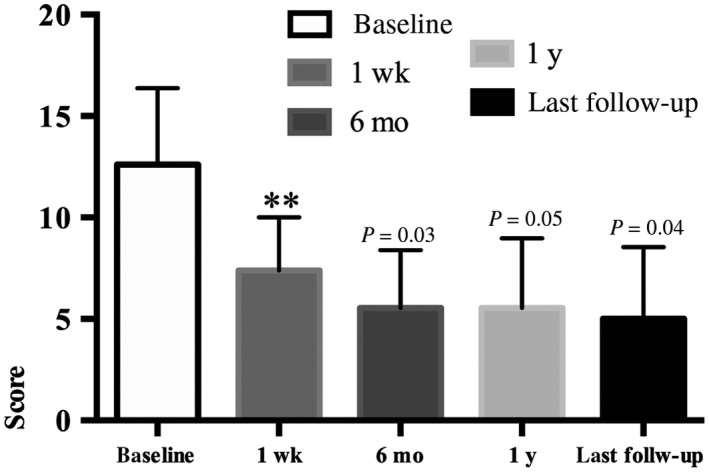
This column bar graph shows the Social Disability Screening Schedule at the postoperative four follow‐ups. **P ≤ 0.001 vs baseline

**Figure 6:**
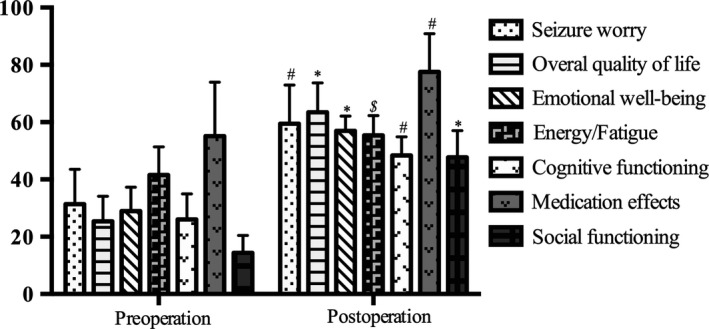
Quality of Life in Epilepsy (QOLIE‐31) subscale scores before surgery and one year after surgery. # indicates *P = *0.02; *indicates *P = *0.01; $ indicates *P = *0.04, as assessed by using Wilcoxon signed‐ranks test

After surgery, the patients' social functioning, as measured by the SDSS, was significantly improved. Social function improved within a week and continued to improve until 6 months, reaching a plateau thereafter (Table 1, Figure 5). Furthermore, all aspects of the patients' quality of life, as indexed by the QoLIE‐31 subscale scores, showed marked improvements at 1‐year follow‐up (Figures 2, 3, 4, 5, 6).

### 3.4 Complications and side effects

Fatigue and laziness were the most common side effects. However, these side effects were transient and disappeared within 1‐4 weeks. As observed in other studies,^13^, ^17^, ^18^ a few patients experienced headache, postoperative confusion, transient memory deficits, or weight gain following surgery. No intracranial hemorrhage or long‐term or fatal complications occurred during the study.

## 4 DISCUSSION

In this study, we explored the utility of bilateral anterior capsulotomy in managing severe psychiatric comorbidities in patients with epilepsy. After anterior capsulotomy, patients displayed significant improvements of psychotic symptoms, as well as reduced levels of impulsivity and aggression, along with improvements of social function and quality of life. These clinical benefits were evident within six months after surgery and remained stable or continued to improve at a slower rate thereafter. Furthermore, no significant side effects or complications occurred during the surgery and follow‐up period. Accordingly, it seems that anterior capsulotomy can offer a safe and effective treatment for epilepsy patients with otherwise intractable psychiatric comorbidities.

Moreover, after surgery all patients complied with epilepsy interventions that they did not comply with prior to surgery. Before anterior capsulotomy, the patients' compliance with the clinical management of their seizures was severely disrupted due to the nature of their comorbid psychiatric symptoms, particularly the presence of pathological suspiciousness about medication plans, agitation, and aggression toward mental health care professionals. Hence, as we hypothesized, it seems that anterior capsulotomy can greatly improve the patients' compliance with epilepsy treatment by alleviating their psychiatric comorbidities.

Anterior capsulotomy was associated with marked improvements of the patients' comorbid psychotic symptoms and aggression. This finding substantiates the results of a recent study of patients with schizophrenia.^15^ Although little is known about the neurobiological mechanisms through which anterior capsulotomy produces its clinical effects, it may be hypothesized that this neurosurgical treatment, similar to antipsychotic medications, can resolve abnormal dopaminergic neurotransmission in the striatum, which mediates the positive symptoms of schizophrenia.^19^, ^20^ In this view, anterior capsulotomy may act not only to restore function of diencephalic and limbic circuits involved in the initiation of emotional responses, such as fear and rage. But anterior capsulotomy may also recover frontal lobe functions that mediate higher cognitive functions and behavioral control.^21^ Regardless of the precise mechanisms involved, it seems that anterior capsulotomy may have a role to play in the treatment of schizophrenia and related psychotic disorders. However, to date, only some but not all studies have reported that anterior capsulotomy is an effective treatment for schizophrenia.^22^ Furthermore, in contrast to pharmacotherapy, anterior capsulotomy is irreversible and a treatment option for only the most severe and otherwise intractable cases.

Finally, we observed that large individual patient differences existed in the clinical response to anterior capsulotomy. For example, the frequency of seizures declined in 11 patients after surgery, but two patients showed no clinical response. These two patients displayed a clinically meaningful response only after having received a second anterior capsulotomy a few years later. It turned out that these patients exhibited more severe psychiatric comorbidity than the other patients at the time of the first surgery, which apparently affected their clinical response to the surgery. Thus, due to large individual differences, a patient‐ and symptom‐centered approach should be utilized to improve the clinical outcome of anterior capsulotomy.

## 5 CONCLUSION

Anterior capsulotomy seems to be an effective treatment for epilepsy patients with otherwise refractory psychotic symptoms and aggression. Moreover, this neurosurgical treatment seems to improve the patients' compliance with anti‐epileptic medication regimens. However, given the relatively small patient sample and observational nature of the study, larger and well‐controlled clinical trials are required before these conclusions can be accepted.

## CONFLICT OF INTEREST

On behalf of all authors, the corresponding author states that there is no conflict of interest.
